# Hyperphagia in Bardet–Biedl syndrome: Pathophysiology, burden, and management

**DOI:** 10.1111/obr.13915

**Published:** 2025-04-04

**Authors:** Philip L. Beales, Metin Cetiner, Andrea M. Haqq, Jennifer Miller, Ashley H. Shoemaker, Diana Valverde, Miriam Zacchia, Hélène Dollfus

**Affiliations:** ^1^ UCL Great Ormond Street Institute of Child Health London UK; ^2^ Children's Hospital, Pediatrics II University of Essen Essen Germany; ^3^ Division of Pediatric Endocrinology University of Alberta Edmonton Alberta Canada; ^4^ Department of Pediatrics University of Florida College of Medicine Gainesville Florida USA; ^5^ Pediatric Endocrinology Vanderbilt University Medical Center Nashville Tennessee USA; ^6^ CINBIO Rare Diseases Group, Universidad de Vigo Galicia Sur Health Research Institute (IIS Galicia Sur) Spain; ^7^ Department of Translational Medical Sciences University of Campania 'Luigi Vanvitelli' Naples Italy; ^8^ CARGO and Department of Medical Genetics Hôpitaux Universitaire de Strasbourg Strasbourg France

**Keywords:** Bardet–Biedl syndrome, hyperphagia, MC4R pathway, obesity

## Abstract

Bardet–Biedl syndrome (BBS) is a rare, genetically heterogeneous, and highly pleiotropic autosomal recessive ciliopathy. Patients typically present with early loss of vision, hyperphagia, severe obesity, learning difficulties, and renal dysfunction. In patients with BBS, dysfunction of the immotile primary cilia in the hypothalamic melanocortin‐4 receptor (MC4R) pathway responsible for controlling energy balance, hunger, and satiety results in severe hyperphagia manifesting in food‐seeking behaviors that drive the development of obesity early in childhood. These behaviors have negative impacts on many areas of the lives of patients with BBS and their families/caregivers, including sleep, mood, school/work, and social/family relationships. Additionally, many patients feel stigmatized due to their hyperphagia‐associated food‐seeking behaviors and the resulting obesity, which exacerbates the impacts of hyperphagia on quality of life. Early identification and management of hyperphagia in patients with BBS is key: mitigating food‐seeking and weight gain can improve quality of life and reduce the risk of metabolic and cardiovascular diseases that is increased in patients with BBS. Until recently, the only treatment strategies available were lifestyle and diet modifications. However, targeted treatment with the novel MC4R agonist setmelanotide now offers an effective management option to reduce hyperphagia and weight in patients with BBS, improving overall health and quality of life.

**Abbreviations**
BBS: Bardet–Biedl syndrome
GLP‐1: glucagon‐like peptide‐1
HQ‐CT: Hyperphagia Questionnaire for Clinical Trials
MC4R: melanocortin‐4 receptor
POMC: pro‐opiomelanocortin
α‐MSH: alpha‐melanocyte‐stimulating hormone

## 1 INTRODUCTION

Bardet–Biedl syndrome (BBS) is a rare, autosomal, recessive ciliopathy^1^, ^2^, ^3^ primarily characterized by retinal (rod–cone) dystrophy, hyperphagia, early‐onset severe obesity, polydactyly, renal dysfunction, hypogonadism, and learning difficulties^1^, ^3^, ^4^, ^5^, ^6^; these characteristics can vary in severity and appearance, and not all of these clinical features are present in every patient. The reported prevalence of BBS ranges from 1:100,000 to 1:160,000 in North America and Europe,^1^, ^3^ but is as high as 1:13,500 to 1:18,000 in some isolated populations, such as the nomadic Bedouins and remote communities in Newfoundland.^7^, ^8^

BBS is a genetically heterogeneous, highly pleiotropic syndrome caused by variants in the genes that encode proteins in the primary cilium/basal‐body complex.^9^ The primary cilium/basal‐body complex influences cell‐to‐cell signaling and is ubiquitously expressed in multiple organs, hence the syndromic nature of BBS.^1^ Variants in at least 26 genes have been recognized in patients with BBS, with another 4 genes classified as modifiers, and this number is increasing.^1^, ^9^, ^10^, ^11^, ^12^, ^13^, ^14^, ^15^, ^16^, ^17^, ^18^ The genotype–phenotype associations in BBS are not yet well understood; patients who have the same genotype often express different symptoms, and a higher mutational load appears to be associated with a more severe phenotype.^1^, ^3^, ^5^, ^17^, ^19^, ^20^, ^21^ Furthermore, evidence is beginning to emerge that suggests that the nature of the causative gene(s) and the type of mutation can be valuable in predicting clinical outcomes in patients with BBS.^10^

In patients with BBS, dysfunction of the immotile primary cilia in the hypothalamic melanocortin‐4 receptor (MC4R) pathway responsible for controlling energy balance, hunger, and satiety appears to be a major contributor to severe pathological hunger, known as hyperphagia, accompanied by food‐seeking behaviors that drive the development of early‐onset severe obesity.^2^, ^5^, ^22^, ^23^, ^24^, ^25^, ^26^, ^27^ Hyperphagia and the resulting severe obesity that develops during childhood contribute to the higher risk of type 2 diabetes and cardiovascular disease seen in patients with BBS, and represent a significant disease‐related health burden that both reduces patient quality of life and has a major impact on the lives of caregivers, parents, and other family members (such as siblings).^28^, ^29^, ^30^

Currently, a comprehensive understanding of the development, role, and burden of hyperphagia in patients with BBS is lacking. This review summarizes the most recent knowledge on the development of hyperphagia in BBS, its impact on patients and their families/caregivers, and recent advances in its treatment.

## 2 HYPERPHAGIA, OBESITY, AND BBS

Early and severe obesity is a cardinal clinical feature of BBS, and obesity is present in between 72% and 92% of all patients with the condition.^1^ Furthermore, based on self‐reported data from a BBS UK survey, 70% of patients living with BBS have obesity.^31^ Another BBS UK survey indicates that, based on the described symptoms, 60% of patients with BBS have severe hyperphagia and the remainder have mild/moderate hyperphagia, with the severity of hyperphagia being defined by expert physicians in the dedicated BBS clinics based on detailed discussions with patients and caregivers.^31^ Assuming that almost all patients with severe hyperphagia have or develop obesity, this indicates that around 86% (60/70) of patients have obesity and severe hyperphagia, and the remaining 14% (10/70) have obesity and mild/moderate hyperphagia. The primary reason for early weight gain in BBS could be associated with severe, persistent hyperphagia that drives food‐seeking behaviors, causing an imbalance between calorie intake and expenditure.^5^, ^30^ As a consequence, in patients with BBS, obesity usually develops early in childhood, with 60% of patients with BBS in the 2–5 year age bracket having obesity and that figure increasing to over 70% in all age groups.^25^ There are conflicting data about the impact of BBS variants on energy expenditure; one study reports that adults with BBS and overweight/obesity have a similar resting metabolic rate – measured using indirect calorimetry – to individuals with overweight/obesity and without BBS, with slightly lower levels of physical activity observed in individuals with obesity with BBS compared with controls.^32^ However, mouse models of BBS to assess energy expenditure have found that BBS mice with obesity have a lower energy expenditure compared with control animals.^33^, ^34^ Future controlled studies using doubly labeled water or whole‐body calorimetry, with considerations for body mass and composition, are therefore needed to determine the impact of energy expenditure on weight gain in individuals with BBS.

Hyperphagia and resulting obesity are some of the earliest symptoms of BBS and should always prompt further clinical investigation; the development of obesity often begins in infancy and persists throughout adulthood.^25^, ^35^ Hyperphagia can be defined as a pathologically increased need for food that is distinct from hunger or appetite; individuals with hyperphagia have persistent, abnormal food‐seeking behavior, abnormal eating patterns, and abnormal or severe preoccupation with food.^30^, ^36^ Overeating behaviors are described as a spectrum ranging from mild to severe, from occasional overeating to feasting, eating without hunger, loss of control overeating, binge eating, and, finally, hyperphagia, the most severe form of overeating behavior.^37^ Characterized by a persistent preoccupation with food, a prolonged time to satiation, shortened duration of satiety, and prolonged feeling of hunger, hyperphagia may justifiably be differentiated from the other overeating behaviors on this spectrum.

### 2.1 Presentation of hyperphagia by age group

Hyperphagia varies in presentation between children and adults. No development trajectory for hyperphagia in BBS has been formally determined, but, in infancy, patients may be fussy feeders, needing feeding every 2 hours and often spitting up due to over‐ingestion of milk. Toddlers with BBS commonly snack very frequently, never seem to be full, and become easily upset if denied food. By school age, the patient will usually display an increased interest in food, talk about food a lot, and try to sneak extra food or steal more snacks.^25^, ^28^, ^29^, ^30^, ^35^, ^37^ For some patients, the severity of their hyperphagia continues as they develop, while others are able to manage their hyperphagia better in adulthood (depending on cognitive impairment); in some patients, hyperphagia may disappear.^28^, ^30^

The Dykens Hyperphagia Questionnaire,^38^ originally developed for patients with Prader–Willi syndrome, has been used to assess hyperphagia in patients with BBS.^5^ Using the Dykens Hyperphagia Questionnaire, a small cohort study of 13 children with BBS reported that hyperphagia is primarily characterized by increased food‐seeking behaviors such as waking up at night to find food, searching trash, and stealing and hiding food.^5^

Recently, the new Symptoms of Hyperphagia Questionnaire was developed and has been used to evaluate the type and frequency of hyperphagia‐associated behaviors observed by caregivers of patients with BBS.^30^ The Symptoms of Hyperphagia Questionnaire contains five items designed to assess the frequency at which caregivers observe hunger‐related behaviors in the patients they are caring for. Caregivers were asked to record how often over the past 24 hours the patient with BBS asked/argued for more food, ate extremely rapidly, took food without permission, woke up at night asking for food, and asked for additional food immediately or shortly after finishing a meal.^30^ The results of this multinational survey of over 200 adult caregivers supported the finding of the smaller study carried out by Sherafat‐Kazemzadeh et al, which concluded that hyperphagia‐associated behavior in BBS is predominantly food‐seeking in nature, with most children with BBS displaying food‐seeking behaviors multiple times each day (Table 1).^5^, ^30^

**TABLE 1:** Symptoms of hyperphagia in patients with BBS reported by their caregivers over the past 24 hours.^26^

Symptom of hyperphagia	Prevalence, % (N = 242)
Negotiates or argues for more food	89.7
Wakes up asking/searching for food during the night	88.4
Asks for more food after just finishing a meal or snack	85.5
Eats extremely quickly	81.4
Sneaks or takes food without permission	80.6

Abbreviation: BBS, Bardet–Biedl syndrome.

## 3 HOW DOES BBS CAUSE HYPERPHAGIA?

**FIGURE 1:**
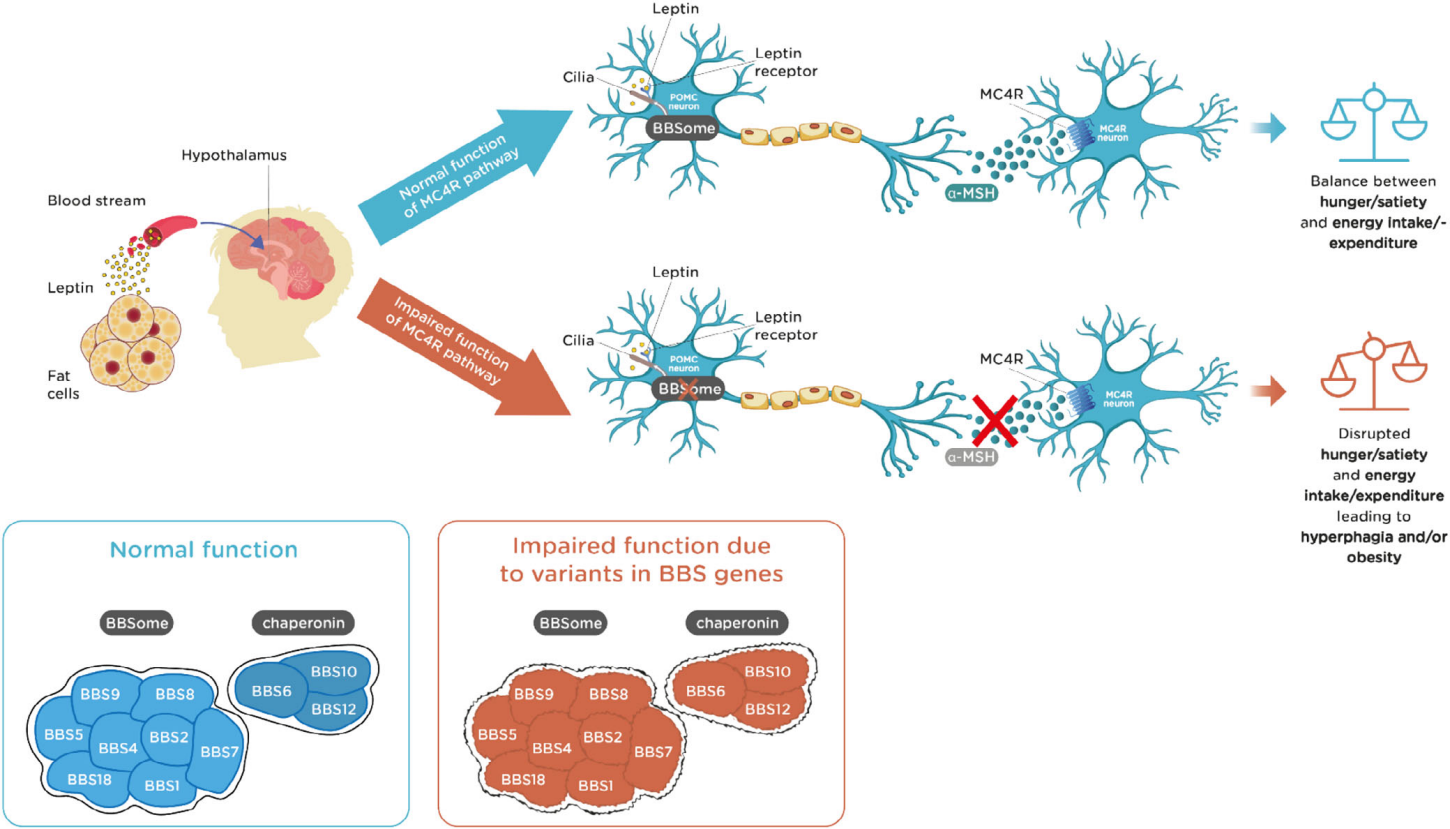
The role of the primary cilia in leptin signaling and control of hyperphagia. The BBSome is involved in cilia function and leptin receptor signaling. Patients with BBS have an impaired MC4R pathway, which can be caused by a variant of one or more of the genes that encode the BBSome proteins (BBS1, BBS2, BBS4, BBS5, BBS7, BBS8, BBS9, BBS18). This can cause both dysfunction of the cilia and disruption of leptin receptor signaling. BBS6, BBS10, and BBS12 are thought to be chaperone proteins involved in BBSome assembly, while the role of other proteins has yet to be determined.^16^ α‐MSH, alpha‐melanocyte‐stimulating hormone; BBS, Bardet–Biedl syndrome; LEPR, leptin receptor; MC4R, melanocortin‐4 receptor; POMC, pro‐opiomelanocortin.

The MC4R pathway regulates the balance between hunger and satiety. Under normal physiological conditions, leptin binds to the leptin receptor on pro‐opiomelanocortin (POMC)‐expressing primary ciliated neurons in the arcuate nucleus of the hypothalamus. The protease encoded by *PCSK1* then cleaves POMC to generate the anorexigenic alpha‐melanocyte‐stimulating hormone (α‐MSH). Proprotein convertase subtilisin/kexin type 1 (PCSK1) cleaves POMC to produce intermediate peptides, including ACTH (adrenocorticotropic hormone), which is then further cleaved by prohormone convertase 2 (PC2) to produce α‐MSH and other peptides. Carboxypeptidase E (CPE) then removes basic amino acids from the C‐terminal end of α‐MSH, peptidyl α‐amidating monooxygenase (PAM) adds an amide group to the C‐terminal end, which is crucial for biological activity, N‐acetyltransferase (N‐AT) acetylates the peptide to affect stability and activity, and prolylcarboxypeptidase (PRCP) further processes the peptide to ensure it is in its final active form.^39^, ^40^ The release of α‐MSH activates MC4R and subsequently reduces hunger and increases energy expenditure.^2^, ^22^, ^41^ In patients with BBS, the MC4R pathway is impaired, which leads to hyperphagia and decreased energy expenditure, resulting in early‐onset severe obesity (Figure 1).

The BBSome is a protein complex that controls ciliary membrane protein composition; it plays a central role in primary ciliary homeostasis and is central to both the proper functioning of the cilia and adequate leptin receptor signaling.^42^ Patients with BBS can have variants in genes encoding the eight protein subunits that form the BBSome, resulting in impaired function of the primary cilia.^9^, ^24^, ^34^, ^42^, ^43^, ^44^ BBS is therefore classed as a ciliopathy. Recently, it has been shown that three of the genes involved in BBS encode chaperone‐like proteins, meaning BBS is also considered a chaperonopathy.^27^, ^43^ Additionally, there are other BBS proteins that are not part of these groups of proteins, but their role is not yet fully elucidated.^16^

Primary cilia are found throughout the body, including in the first‐order neurons in the arcuate nucleus of the hypothalamus, which regulates energy balance, hunger, and satiety.^2^, ^24^, ^34^, ^42^ As mentioned previously, leptin is an anorexigenic hormone, suppressing hunger.^2^ Therefore, disrupted leptin signaling in patients with BBS results in increased hunger and reduced satiety, which induces hyperphagia. Mouse models of BBS have demonstrated leptin resistance in the hypothalamic MC4R pathway, which drives hyperphagia and obesity and may be explained by impaired leptin signaling in the hypothalamic neurons resulting from disrupted functioning of the neuronal cilia.^33^, ^42^ Cellular models of BBS with *BBS1* and *BBS10* variants have demonstrated reduced leptin signaling in hypothalamic neurons, which can be restored by correcting the genetic alterations,^27^ and genetic studies in mice have shown that primary cilia, specifically on MC4R‐expressing neurons, are required for control of energy metabolism.^26^ Although there are limited data from human studies, a cohort study of 50 patients has shown that patients with BBS have increased levels of leptin and triglycerides and higher diastolic blood pressure compared with age‐, sex‐, and race‐matched controls.^20^ Therefore, it appears that one key dysfunction caused by BBS ciliary‐protein variants in patients with BBS is impaired leptin receptor signaling, which disrupts the hypothalamic leptin–MC4R pathway that governs energy regulation, resulting in the hyperphagia observed in BBS.^2^, ^22^, ^23^, ^24^, ^26^, ^27^, ^34^

## 4 BURDEN OF HYPERPHAGIA IN BBS

Individuals with self‐reported hyperphagia and severe obesity have a lower quality of life compared with the general population.^45^ Alongside the distressing impact of the severe visual impairment experienced by almost all patients with BBS, children with BBS and their parents/caregivers have reported that hyperphagia and the resulting severe obesity are two of the most distressing features of the disease and are often associated with negative perceptions of the patient and their families.^46^ The societal impacts of hyperphagia in patients with BBS are only just beginning to be understood and characterized. Quality of life, relationships, productivity, school performance, and emotions are among the key areas that patients and their families/caregivers report as being negatively affected (Table 2).^28^, ^29^, ^30^, ^36^, ^46^

**TABLE 2:** Summary of key impacts of hyperphagia on patients with BBS and their caregivers.

Impact of hyperphagia	Patients with BBS	Parents/caregivers
Impaired quality of life ^24^	✓	
Impaired concentration ^26^, ^32^	✓	
Impaired relationships ^25^, ^26^, ^32^	✓	✓
Negative emotions (sadness, frustration, guilt) ^25^, ^26^, ^32^, ^41^	✓	✓
Impaired productivity at school or work ^25^, ^26^, ^32^	✓	✓
Impaired sleep ^25^, ^26^	✓	✓
Courtesy stigma ^41^		✓
Financial burden ^25^		✓

Abbreviation: BBS, Bardet–Biedl syndrome.

### 4.1 Impact on patients

Hyperphagia has been reported to have a negative impact on many aspects of patients' lives. Children with BBS can be stigmatized as a result of their hyperphagia and may struggle to establish friendships in their peer group at school due to their extreme food‐seeking/eating behaviors and early obesity.^46^ A cross‐sectional multinational survey of caregivers of patients with BBS (CAREgiver Burden in BBS survey; CARE‐BBS) recently reported the responses from 242 caregivers of patients with BBS^30^ and found that hyperphagia had either a moderate or great impact on sleep in 54% of patients, on mood/emotions in 56% of patients, on school in 57% of patients, on leisure in 62% of patients, and on family relationships in 51% of patients (Table 3).^30^ Hyperphagia affects school performance because school‐age patients with BBS have difficulty maintaining concentration^30^, ^36^ and a high proportion regularly miss at least 1 day of school a week, which subsequently also affects the lives of their parents/caregivers.^30^ Furthermore, the obesity that results from hyperphagia has been identified as having a negative impact on the patient's body image/self‐esteem, social life, and physical comfort.^30^

**TABLE 3:** Results of the Impacts of Hyperphagia Questionnaire in patients with BBS.^26^

Impacts of hyperphagia (% of respondents affected)	Patients with BBS^a^	Caregivers
***Sleep***		
Not at all affected	7.0%	9.5%
A little affected	39.3%	33.9%
Moderately affected	39.7%	38.8%
A great deal affected	14.0%	17.8%
***Mood/emotion***		
Not at all affected	14.5%	11.6%
A little affected Moderately affected	29.8% 35.1%	31.8% 40.1%
A great deal affected	20.7%	16.5%
***School/work***		
Not at all affected	10.7%	13.6%
A little affected	32.6%	33.1%
Moderately affected	40.9%	38.0%
A great deal affected	15.7%	15.3%
***Leisure***		
Not at all affected	12.8%	14.0%
A little affected	25.6%	33.1%
Moderately affected	42.1%	33.5%
A great deal affected	19.4%	19.4%
***Familial relationships***		
Not at all affected	12.4%	16.5%
A little affected	36.8%	35.5%
Moderately affected	37.2%	34.3%
A great deal affected	13.6%	13.6%

Abbreviation: BBS, Bardet‐Biedl syndrome.

^a^ 97% of patients were <18 years of age.

A recent patient and caregiver interview‐based study by Ervin et al reported that BBS‐associated uncontrollable hunger is commonly associated with obsessive food‐seeking and overconsumption of food, with limited, short‐lived feelings of satisfaction following eating in patients with BBS.^36^ This study also found that hyperphagia impacted patients' emotions, resulting in feelings of anxiety and irritability, which subsequently affected their relationship with parents/caregivers and other family members, most specifically by causing tensions with parents/caregivers and impairing relationships with siblings.^36^

Patients with BBS have been shown to be at higher risk for a number of comorbid conditions, including insulin resistance, metabolic syndrome, type 2 diabetes, hypertension, and cardiovascular disease.^20^, ^47^ It has also been shown that patients with BBS have higher levels of triglycerides, higher fasting blood glucose, and higher blood pressure than age‐matched controls,^20^, ^47^ and it has been suggested that the intra‐abdominal visceral pattern of fat deposition may predispose patients with BBS to metabolic complications such as type 2 diabetes.^20^ Due to the multisystemic effects of BBS variants on the primary cilia, the risk of these conditions is greater in patients with BBS than would be expected from the effects of obesity alone.^20^, ^47^ However, mitigating hyperphagia would help to control weight gain, and thereby help to prevent the development (and assist in the management) of metabolic syndrome, type 2 diabetes, and cardiovascular disease.^35^ In addition to visual impairment, severe obesity in patients with BBS is likely to lead to reduced mobility, though this has not been directly studied to date. Therefore, reducing hyperphagia and obesity may also facilitate patients with BBS in becoming more physically active.

### 4.2 Impact on parents/caregivers

Hyperphagia also has a significant impact on the lives of parents/caregivers of patients with BBS. This has been understudied to date, but recent interview‐ and survey‐based studies have reported a significant burden of hyperphagia on parents/caregivers. The recent CARE‐BBS survey found that adult caregivers of young patients with BBS deal with the associated hyperphagia throughout the day and night, with 90% of patients regularly negotiating for more food than provided, and 88% regularly waking at night and asking for food.^30^ Using a version of the new Impacts of Hyperphagia Questionnaire specifically designed for caregivers, the CARE‐BBS survey also identified that – in a similar pattern to that seen in patients themselves – the hyperphagia of children with BBS had moderate‐to‐severe negative impacts on caregivers' sleep (57%), mood/emotions (57%), work (53%), leisure and recreation (53%), and relationships with friends and family (48%).^29^ Caregivers (mainly comprised of parents of patients) who remain in the workforce report impaired ability to work due to caring for their patients with BBS, and more than half of the 242 respondents in CARE‐BBS reported having significant out‐of‐pocket expenditure to cover medical costs for the patient with BBS in their care.^29^ Similar findings were reported in a patient and caregiver interview‐based study of hyperphagia in patients with BBS^36^; in addition, caregivers reported that food‐seeking behavior in the patients they care for affects them emotionally, increasing feelings of anxiety, depression, and helplessness.^36^ Hyperphagia associated with BBS also impacts other family members living with the patient. The impact of hyperphagia on siblings of patients with BBS is an area that has not been well studied to date, but it is to be expected that a sibling with hyperphagia will impact other children in a family both due to the direct effects on mood and relationships, as highlighted in the study by Ervin et al,^36^ and through more indirect effects such as sibling jealousy and rivalry for food and parent/caregiver attention. Relationships between couples, and between parents and other family members, can also become strained, and parents report anger, frustration, sadness, and depression as they try to manage food intake for their child.^36^, ^46^

Patients with BBS struggle to live independently as adults due to their visual impairment, cognitive impairment/learning difficulties, and hyperphagia. As a consequence, parents who have a child with BBS expect to continue to care for their child into adulthood. This indefinite care burden can lead to exhaustion among parents/caregivers – particularly as the child with BBS reaches adulthood, as it is much more difficult to control and manage access to food for adults with BBS. There is therefore a need for specialized group‐home settings with experience in managing hyperphagia and locked food storage, but such care settings can be difficult to find for patients with BBS.

Parents/caregivers have also reported feelings of courtesy stigma – stigma experienced because of close association with another person – in relation to their communication with healthcare professionals and surrounding their child with BBS, perceiving that they are being blamed or judged for the child's obesity and for being unable to control the patient's food‐seeking behavior and weight gain.^46^, ^48^ However, in reality, parents/caregivers are very aware that hyperphagia has multiple potential health consequences for their child with BBS, and report feelings of guilt and failure because they are unable to prevent the development of obesity.^36^ Courtesy stigma and feelings of being misunderstood can impede effective communication between parents and the healthcare team surrounding the management of hyperphagia.^46^ In addition, nursery staff and schoolteachers usually lack information about BBS as a disease^48^ and do not receive training on how to manage hyperphagia and its behavioral consequences appropriately, which can exacerbate feelings of frustration and helplessness in parents/caregivers.

## 5 MANAGEMENT OF HYPERPHAGIA IN BBS

Since BBS affects multiple organ systems, patients with BBS require a multidisciplinary management approach with regular clinical review.^3^, ^18^ The multidisciplinary management team should include a geneticist, pediatrician, nutritionist/dietician, gastroenterologist, endocrinologist, nephrologist, ophthalmologist, dentist, psychologist, and speech and language therapist, plus a nurse and patient support group representative.^1^, ^6^ Regular developmental and educational assessments are important and, if needed, referrals can be made to an orthodontist, clinical psychologist, audiologist, or cardiologist.^3^

Management of hyperphagia should be at the center of the patient care plan for BBS. Hyperphagia management should be a particular focus of care in young patients with BBS, as improving hyperphagia is key to reducing obesity and preventing future complications such as type 2 diabetes and cardiovascular disease, as well as preventing mobility issues, mitigating mental health concerns, and improving quality of life for both patients and their families/caregivers. Identifying hyperphagia early is key, and it is important to be aware that the presentation of hyperphagia changes over time as the child with BBS develops. Hyperphagia in patients with BBS has been assessed using the Dykens Hyperphagia Questionnaire,^5^, ^38^ originally developed for use with patients with Prader–Willi syndrome. However, the Dykens Hyperphagia Questionnaire has several important limitations (e.g., it does not lend itself to accuracy in food‐secure environments and does not quantify the number of each type of hyperphagia‐associated behavior/day). The more recently developed Symptoms of Hyperphagia Questionnaire is a more tailored instrument for the assessment of hyperphagia in patients with BBS,^30^ but it is important to note that, in both cases, the questionnaires need to be administered in person, with the caregiver present, which limits their use. Another potential limitation is the ability of questionnaires to accurately assess the degree of hyperphagia in patients in an environment where access to food is highly controlled. The Hyperphagia Questionnaire for Clinical Trials (HQ‐CT) was developed by adapting the Dykens Hyperphagia Questionnaire, and an analysis of the HQ‐CT in patients with Prader–Willi syndrome^49^ showed that patients with a strict food routine and carefully restricted food access had lower HQ‐CT scores than other patients with Prader–Willi syndrome. Another approach could be the inclusion of potential biomarkers to measure hunger and satiety (e.g., short‐term regulators of food intake such as ghrelin or GLP‐1 or long‐term regulators such as leptin). Limitations with the use of biomarkers are that measurement of one biomarker is not sufficient to assess hunger and satiety, and high inter‐individual variability of the biomarkers makes it difficult to compare between subjects.^50^, ^51^, ^52^A different approach is required to assess hyperphagia symptoms in adults, but no adult‐specific tools currently exist.

Until recently, the treatment strategies for managing obesity in patients with BBS were based on modified diet and/or increased physical activity plans. However, many patients with BBS struggle to cope with or adhere to lifestyle modifications, possibly due to their extreme hyperphagic drive^46^; therefore, changes to lifestyle and nutrition (alone or with exercise interventions) are unlikely to be successful unless the patient's underlying hyperphagia is specifically addressed.

## 6 ROLE OF PHARMACOTHERAPY IN THE HYPERPHAGIA OF BBS

Currently, the only approved medication for chronic weight management and control of hyperphagia in patients with BBS is the novel MC4R agonist setmelanotide.^53^ Setmelanotide is an analog of MSH, which works by restoring signaling through the MC4R pathway, leading to a satiety signal, decreased hyperphagia, increased energy expenditure, and subsequent weight loss.^54^ Phase 2 and 3 clinical studies have shown that targeted treatment with setmelanotide can reduce hunger and body weight in patients with BBS^30^, ^54^, ^55^; clinically meaningful improvements in quality of life have also been reported following 1 year of treatment with setmelanotide in children and adults with BBS.^28^, ^36^ Setmelanotide therefore represents a valuable new treatment for hyperphagia and obesity in patients with BBS.

Besides the MC4R‐targeted treatment, glucagon‐like peptide‐1 (GLP‐1) receptor agonists (such as semaglutide) in combination with lifestyle interventions can reduce body weight in individuals with overweight or obesity.^56^ A case study of a young woman with BBS has reported that treatment with GLP‐1 receptor agonists plus a lifestyle intervention led to decreased body weight.^57^ However, the effect of GLP‐1 receptor agonists on body weight in patients with BBS during a randomized, double‐blind, placebo‐controlled clinical trial remains to be determined. In addition, despite the availability of earlier‐generation GLP‐1 agonists (e.g., liraglutide from 2014/2015) and significant unmet medical need, there are limited reports and no randomized clinical trials.

Other new anti‐obesity drugs, such as the glucose‐dependent insulinotropic polypeptide and GLP‐1 receptor agonist tirzepatide,^58^ have the potential to be effective for managing obesity in patients with BBS and may be valuable in combination with setmelanotide in the future.

Bariatric surgery is another treatment option; however, a 15‐year case–control study has shown that, for patients with genetic mutations affecting the leptin–melanocortin pathway and who undergo Roux‐en‐Y gastric bypass, weight is lost in the short term but there is significant regain in the long term.^59^ Retrospective studies in adult patients have shown benefits in the use of GLP‐1 receptor agonists following bariatric surgery, allowing weight regain to be reduced.^60^, ^61^ This highlights the need for clinical trials examining the combination of bariatric surgery and pharmacotherapy in order to allow patients with an impaired leptin–melanocortin pathway to derive long‐term benefits from bariatric surgery.

## 7 CONCLUSIONS

Hyperphagia and the resulting early‐onset severe obesity place a significant physical and psychosocial burden on patients with BBS and significantly impact the lives of their families/caregivers. It is important that hyperphagia is recognized early and routine monitoring is considered, particularly in children and adolescents, for early identification of patients with BBS.^18^ In young patients with BBS, hyperphagia manifests as food‐seeking behaviors. If not managed, it can lead to severe obesity, worsening metabolic issues and increasing the risk of diabetes and cardiovascular disease, impacting both short‐ and long‐term health. Additionally, hyperphagia affects quality of life, impacting sleep, mood, social life, education, and family relationships. It also burdens caregivers, causing frustration, anxiety, and depression, and has financial consequences due to health expenses and reduced work productivity. Until recently, the only management options for hyperphagia in BBS were dietary modifications and lifestyle/exercise plans, both of which are difficult to implement due to patients' learning difficulties and the severity of food‐seeking behaviors in non‐food‐secure environments, such as home and school. Targeted treatment with the MC4R agonist setmelanotide offers – for the first time – an effective management option to reduce hyperphagia, supporting weight loss and improvements in quality of life for patients with BBS. The emergence of a disease‐specific treatment option offers significant hope for improved management of hyperphagia and obesity in BBS. However, there are a number of important areas of research that need to be addressed in order to improve future management of hyperphagia and obesity in BBS, including the development of new tools to assess hyperphagia (in adults with BBS, for example) and the evaluation of treatment regimens using setmelanotide in combination with other novel agents such as tirzepatide and the combination of bariatric surgery and pharmacotherapy to prevent excessive weight regain post‐surgery.

In conclusion, early recognition of hyperphagia and its impacts on BBS, along with subsequent effective management using disease‐specific setmelanotide treatment or a potential combination of therapies, has the potential to significantly improve long‐term health in patients with BBS and to improve quality of life for both patients with BBS and their families/caregivers.

## CONFLICT OF INTEREST

**PLB** has participated on an advisory board for BBS UK; has participated in a webinar and symposium hosted by Rhythm Therapeutics; and serves as CEO of Axovia Therapeutics, a BBS‐focused gene therapy company. **MC** has served as a principal investigator for Rhythm Pharmaceuticals; has received funding from the German Federal Ministry of Education and Research (BMBF); has received consulting fees from Rhythm Pharmaceuticals; and has received honoraria from Alexion, Canon Medical Systems, Desitin, and Rhythm Pharmaceuticals. **AMH** has served as a principal nvestigator for Acadia Pharmaceuticals, Eli Lilly, Levo Therapeutics, and Rhythm Pharmaceuticals; has received grant funding from the Canadian Institutes of Health Research (CIHR), Weston Family Foundation, and Women and Children's Health Research Institute (WCHRI); has received consulting fees from Novo Nordisk Canada, Pfizer Canada, and Rhythm Pharmaceuticals; has received honoraria from Rhythm Pharmaceuticals; and has participated on an advisory board for Novo Nordisk Canada and Rhythm Pharmaceuticals. **JM** has received research funding from Harmony Biosciences, Rhythm Pharmaceuticals, and Soleno Therapeutics. **AHS** has received grants from Rhythm Pharmaceuticals and Soleno Therapeutics; has received consulting fees from LG Chem Ltd, Radius Health, Inc., Rhythm Pharmaceuticals, and Saniona A/S; and has received honoraria from Rhythm Pharmaceuticals. **DV** has received honoraria and support for attending meetings and/or travel from Rhythm Pharmaceuticals. **MZ** has received support for attending meetings and/or travel from Rhythm Pharmaceuticals. **HD** has received consulting fees from Gensight, Novartis, Rhythm Pharmaceuticals, and Sparrington.
